# The clinical characteristics of patients with pulmonary hypertension combined with obstructive sleep apnoea

**DOI:** 10.1186/s12890-021-01755-5

**Published:** 2021-11-21

**Authors:** Lu Yan, Zhihui Zhao, Qing Zhao, Qi Jin, Yi Zhang, Xin Li, Anqi Duan, Qin Luo, Zhihong Liu

**Affiliations:** Center for Pulmonary Vascular Diseases, Fuwai Hospital, National Clinical Research Center for Cardiovascular Diseases, National Center for Cardiovascular Diseases, Chinese Academy of Medical Sciences and Peking Union Medical College, 167 BeiLiShi Road, Xicheng District, Beijing, 100037 China

**Keywords:** Pulmonary hypertension, Obstructive sleep apnoea, Risk factors

## Abstract

**Objective:**

Obstructive sleep apnoea (OSA) is one cause of pulmonary hypertension (PH) and can also emerge along with PH. The clinical diagnosis and treatment of OSA in patients with PH are still controversial. The purpose of this clinical observation study was to observe and summarize the incidence and clinical characteristics of OSA in patients with PH and to explore possible predictors of PH combined with OSA.

**Methods:**

Patients with PH diagnosed by right heart catheterization who underwent overnight cardiorespiratory monitoring from December 2018 to December 2020 were enrolled. OSA was defined as an apnoea–hypopnoea index of ≥ 5/h with ≥ 50% of apnoeic events being obstructive. Baseline clinical characteristics and parameters were collected to compare PH patients with and without OSA. Logistic regression analysis was run to determine the risk factors for OSA in PH patients.

**Results:**

A total of 35 (25%) of 140 patients had OSA. OSA is relatively frequent in patients with PH, especially in patients with chronic thromboembolic pulmonary hypertension and patients with lung disease– or hypoxia-associated PH. The patients who had OSA were mostly male and had a higher age and a lower daytime arterial oxygen pressure. Logistic regression analysis found that older age, male sex, and lower daytime arterial blood oxygen pressure correlated with OSA in PH patients.

**Conclusion:**

OSA is common in patients with PH. Lower daytime arterial oxygen pressure is a risk factor for OSA in older male patients with PH.

## Study background

Pulmonary hypertension (PH) refers to a clinical syndrome involving pulmonary vascular structural and functional changes caused by various aetiologies, leading to a progressive increase in pulmonary vascular resistance and eventually causing right heart failure or even death. Its aetiology is complex, and the clinical symptoms are diverse, with a high rate of misdiagnosis and mortality [1]. PH is characterized by a mean pulmonary artery pressure (mPAP) ≥ 25 mmHg (1 mmHg = 0.133 kPa) [1] measured by right heart catheterization (RHC) at sea level and at rest. The mPAP of a normal adult at rest is 14.0 ± 3.3 mmHg, and its upper limit does not exceed 20 mmHg [2]. Critical PH was once defined as mPAP = 21–24 mmHg [3]. At the 6th World Symposium on Pulmonary Hypertension (WSPH) in 2018, some experts suggested that the PH haemodynamic diagnostic criteria should be revised to mPAP > 20 mmHg [4], but due to controversy about the cut-off points, there is still a lack of relevant studies on patients with mPAP between 21 and 24 mmHg in China. Therefore, this guideline does not adopt this diagnostic criterion [5].

According to pathological manifestations, haemodynamic characteristics, and clinical diagnosis and treatment strategies, PH is divided into five categories: ① pulmonary arterial hypertension (PAH); ② pulmonary hypertension caused by left heart disease; ③ pulmonary hypertension caused by hypoxia and/or lung disease; ④ chronic thromboembolic pulmonary hypertension; and ⑤ pulmonary hypertension caused by multiple mechanisms and/or unknown mechanisms. In recent decades, advances in targeted drug therapy have greatly improved the prognosis of PAH. Because of improved diagnostic methods, the number of patients diagnosed with PH has significantly increased. The United States Registry to Evaluate Early and Long-term pulmonary and Arterial Hypertension Disease Management (REVEAL registry) reported that the 5-year survival rate of patients with PAH was still only 61.2% [6].

Obstructive sleep apnoea (OSA) is characterized by recurrent episodes of partial or complete collapse of the upper airway during sleep, resulting in reduced (hypopnoea) or absent airflow (apnoea) that lasts for at least 10 s and is associated with either cortical arousal or a fall in blood oxygen saturation [7]. OSA, which is present in approximately 25% of adults in the United States, is the leading cause of excessive daytime drowsiness, as well as a cause of reduced quality of life, impaired job performance, and increased risk of motor vehicle collisions [7].

OSA is associated with an increased incidence of hypertension, type 2 diabetes mellitus, atrial fibrillation, heart failure, coronary heart disease, stroke, and death [8, 9]. OSA can be diagnosed by either home- or laboratory-based sleep testing, and effective treatments are available. Dumitrascu et al. studied 169 patients with precapillary PH and found 27 patients with OSA [10]. OSA is very common in patients with PH and is associated with disease progression, but the mechanism linking them remains unclear [11–13].

OSA is more common in patients with than without PH. It is currently believed that OSA can cause precapillary PH, which belongs to category III PH, or it can exist as a comorbidity of PH, and the combination of the two has a poor prognosis. In the past, it was generally believed that OSA was mechanistically related to PH, but PH was only seen in a small number of patients with OSA, and their degree of correlation was generally mild. Therefore, OSA is often overlooked in the diagnosis, risk stratification, and treatment of PH. Additionally, there is a lack of research on the relationship between OSA and PH in China. We designed this single-centre study to understand the incidence and clinical characteristics of OSA in patients with pulmonary hypertension in China.

## Study methods

This single-centre observational study was conducted at Fuwai Hospital, National Center for Cardiovascular Diseases, Chinese Academy of Medical Sciences. The study was conducted with the approval of the Ethics Committee of Fuwai Hospital. Written informed consent was obtained from all the participants.

### Study sample

**Fig. 1:**
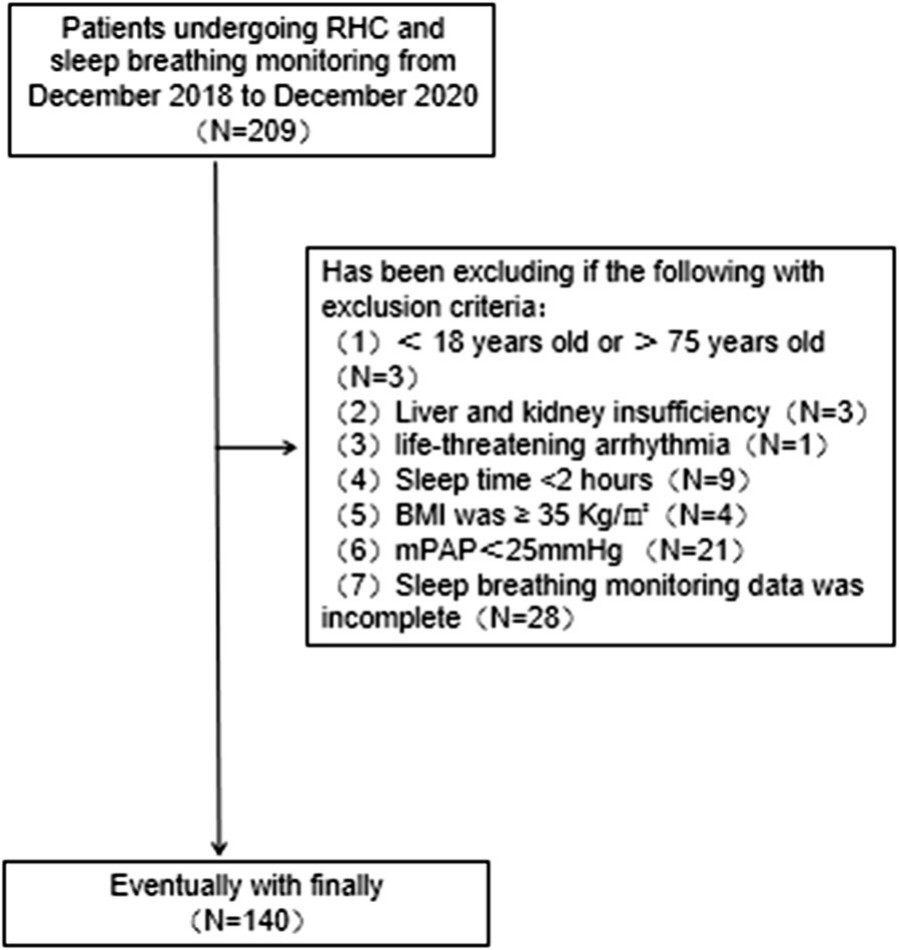
Flow chart of patient inclusion and exclusion. RHC, right heart catheterization; liver and kidney insufficiency, defined as a liver enzyme more than 3 times the normal value and a creatinine clearance rate < 30 ml/min; BMI, body mass index; mPAP, mean pulmonary artery pressure

All enrolled patients underwent respiratory polygraphy and presented a stable clinical state. Respiratory polygraphy examinations were performed before or after RHC at intervals not exceeding 7 days. Data were collected from consecutive hospitalized patients diagnosed with PH by RHC between December 2018 and December 2020. Patients whose RHC results showed a mean pulmonary arterial pressure (mPAP) of ≥ 25 mmHg were diagnosed with PH. Based on the respiratory polygraphy results, the participants were divided into two groups: PH patients without OSA and PH patients with OSA. The exclusion criteria were as follows: (1) patients who were not diagnosed with PH; (2) patients aged < 18 years or > 75 years; (3) patients with chronic liver or renal insufficiency, defined as a liver enzyme more than 3 times the normal value and a creatinine clearance rate < 30 ml/min; (4) patients with life-threatening arrhythmia; (5) patients with nightly sleep time less than two hours; (6) patients with BMI ≥ 35 kg/m^2^; and (7) patients with a previous diagnosis of sleep-disordered breathing (see Fig. 1).

### Patient assessment

The baseline clinical characteristics of each subject, including age, sex, BMI, smoking history, six-minute walk distance (6MWD), World Health Organization functional class (WHO FC), comorbidities, and medication history, were collected separately. In addition, fasting venous blood was collected on admission to evaluate N-terminal pro-brain natriuretic peptide (NT-proBNP), C-reactive protein (CRP) and high-sensitivity C-reactive protein (hs-CRP) levels. Oxygen partial pressure (PaO2), carbon dioxide partial pressure (PaCO2), and peripheral capillary oxygen saturation (SpO2) were determined by arterial blood gas tests. All patients completed the Epworth Sleepiness Scale (ESS) [14] before undergoing sleep breathing monitoring. Echocardiography and pulmonary function testing were performed on each patient before RHC. All subjects underwent RHC to obtain their baseline haemodynamic parameters, i.e., mean right atrial pressure (mRAP), mPAP, cardiac index (CI), and pulmonary vascular resistance (PVR).

### Cardiorespiratory study

Each enrolled PH patient underwent overnight cardiorespiratory recording using an Embletta system (Medcare Flaga, Reykjavik, Iceland) at the sleep centre of Fuwai Hospital. The device recorded nasal airflow, finger pulse oximetry, thoracoabdominal movements, body position, and snoring. According to the American Academy of Sleep Medicine (AASM) Manual for Scoring Sleep and Related Events, sleep apnoea is defined as a complete cessation of oronasal respiratory airflow during sleep or a decrease of more than 90% from baseline, either one lasting for more than 10 s. Hypopnoea is defined as a decrease in respiratory airflow intensity during sleep of more than 30% from baseline, accompanied by a decrease in oxygen saturation ≥ 3% from baseline. If the apnoea-hypopnoea index (AHI) was ≥ 5/h, sleep apnoea was diagnosed. If the apnoea event was accompanied by the cessation of respiratory movement in the chest and abdomen, it was considered a central apnoea event; otherwise, it was considered an obstructive event. OSA was diagnosed if obstructive AHI totalled ≥ 5/h and the obstructive events accounted for > 50% of the apnoeic events. In our study, there were 35 patients diagnosed with OSA among the 140 PH patients. The incidence of OSA was 76%, 50%, 15.4%, 11.8%, and 8% in patients with PH associated with pulmonary disease or low oxygen, CTEPH, CTD-PAH, others, and CHD-PAH, respectively.

### Cardiopulmonary exercise test

All consecutively enrolled patients with newly diagnosed IPAH underwent cardiopulmonary exercise testing (CPET) (Cosmed S.R.L., Rome, Italy). The operational criteria for the cardiopulmonary exercise test were as follows: 3 min of rest, then 3 min of idling, followed by increments of 5–20 W (depending on the patient's exercise capacity) until the patient reached a symptom-limited maximal exercise state. Peak oxygen uptake (peak VO2) was defined as the maximal oxygen uptake over a 30-s period at maximal exertion during the continuous incremental power test. Minute ventilation/carbon dioxide output at the anaerobic threshold (VE/VCO2) was defined as the ratio of required ventilation (VE) per 1 L of CO2 expelled at the anaerobic threshold.

### Statistical analysis

The continuous variables are presented as means ± standard deviations or percentages, and the categorical variables are presented as counts or percentages. For the continuous variables, the means of two independent samples conforming to a normal distribution were compared by the two-independent-samples t-test, and categorical variables were compared by the chi square test. A P-value < 0.05 was considered significant. A logistic regression analysis was also used to explore the factors associated with prolonged OSA in PH patients. All data were analysed by SPSS 22.0 software.

### Basic characteristics of patients included in this study

A total of 164 patients were newly diagnosed with PH and presented a stable clinical status. At the time of this study, they were in stable condition and had been on the same therapy for at least 1 month. Among them, 150 patients underwent overnight cardiorespiratory monitoring before or after RHC. Finally, a total of 140 patients with complete sleep data were enrolled. The baseline clinical characteristics of all PH patients with and without OSA were compared, as shown in Table 1; the average age of the 140 patients was 39.5 ± 13.55 years, and 76.4% (107/140) were female. The average BMI was 23.17 ± 3.86 kg/m2. The average heart rate was 83.42 ± 13.82/min, the average systolic pressure was 113.17 ± 13.94 mmHg, and the average diastolic pressure was 73.4 ± 11.45 mmHg. The mPAP in the static state was 55.73 ± 17.06 mmHg and the average NT-proBNP was 1260.35 ± 1364.62 pg/ml. There were 35 patients diagnosed with OSA among the 140 PH patients. The incidence of OSA was 76%, 50%, 15.4%, 11.8%, and 8% in patients with PH associated with pulmonary disease or low oxygen, CTEPH, CTD-PAH, others, and CHD-PAH, respectively (see Tables 1 and 2).

**Table 1:** Baseline characteristics and 6MWD of all patients

Variable	All patients	IPAH	CTD-PAH	CHD-PAH	Pulmonary disease or low oxygen	CTEPH	unknown mechanisms PH
Number of patients	140	40	13	25	25	20	17
Age (years)	39.5 ± 13.55	30.95 ± 8.91	41.69 ± 11.65	36 ± 14.12	46.28 ± 14.81	48.85 ± 10.27	41.94 ± 12.33
Females (N%)	107 (76.4%)	31 (77.5%)	13 (100%)	21 (84%)	18 (72%)	10 (50%)	12 (71%)
BMI (kg·m^−2^)	23.17 ± 3.86	22.58 ± 3.17	23.62 ± 3.56	20.50 ± 2.88	25.4 ± 4.55	24.76 ± 2.79	23 ± 4.41
SBP (mmHg)	113.17 ± 13.94	110.93 ± 13.03	111.15 ± 18.56	111.4 ± 16.60	115.56 ± 12.91	118 ± 11.22	113.41 ± 12.06
DBP (mmHg)	73.4 ± 11.45	71.95 ± 9.06	73 ± 10.82	72.44 ± 10.34	75.76 ± 9.37	80.55 ± 11.10	66.65 ± 16.88
HR (beats/min)	83.42 ± 13.82	80.6 ± 13.45	82.54 ± 14.12	83.36 ± 14.91	87.2 ± 12.61	86.65 ± 15.56	81.47 ± 12.25
6MWD (m)	405.66 ± 105.08	439.95 ± 104.53	431.11 ± 84.96	435.57 ± 75.19	354.72 ± 87.99	350.11 ± 116.21	404.69 ± 120.54
OSA (N%)	35 (25%)	0 (0%)	2 (15.4%)	2 (8%)	19 (76%)	10 (50%)	2 (11.8%)

Continuous variables are presented as mean ± SD. Categorical variables are given as counts. IPAH, idiopathic pulmonary arterial hypertension; CTD-PAH, pulmonary arterial hypertension associated with connective tissue disease; CHD-PAH, pulmonary arterial hypertension associated with congenital heart disease; CTEPH, chronic thromboembolic pulmonary hypertension; BMI, body mass index; SBP, systolic blood pressure; DBP, diastolic blood pressure; HR, heart rate; 6MWD, six-minute walking distance; OSA, obstruct sleep apnoea

**Table 2:** Baseline blood gas, NT-proBNP, haemodynamics, and CPET examination characteristics of all patients

Variable	All patients	IPAH	CTD-PAH	CHD-PAH	Pulmonary disease or low oxygen	CTEPH	unknown mechanisms PH
Number of patients	140	40	13	25	25	20	17
PaO_2_ (mmHg)	69.75 ± 14.52	75.40 ± 13.13	73.64 ± 12.28	65.51 ± 18.38	65.03 ± 11.94	61.22 ± 7.97	77.08 ± 13.85
PaCO_2_ (mmHg)	35.93 ± 6.89	35.48 ± 3.42	33.64 ± 3.83	36.68 ± 13.19	38.65 ± 13.19	33.73 ± 3.85	36.05 ± 2.70
SaO_2_ (%)	92.88 ± 4.53	94.22 ± 4.76	94.3 ± 2.31	90.95 ± 5.70	91.69 ± 4.43	91.49 ± 3.45	95.09 ± 2.03
NT-proBNP (pg/ml)	1260.35 ± 1364.62	1063.88 ± 1410.25	1527.68 ± 1583.17	1688.02 ± 671.05	1621.12 ± 1188.38	1986.57 ± 1926.25	974.92 ± 769.72
Mean PAP (mmHg)	55.73 ± 17.06	57.55 ± 19.93	50.77 ± 13.52	68.12 ± 20.46	52.76 ± 9.32	48.3 ± 8.9	50.12 ± 13.62
PVR (dyn•s•cm-5)	1128.66 ± 490.40	1214.71 ± 556.99	1032.50 ± 406.93	1077.91 ± 514.50	1298.43 ± 492.18	1008.06 ± 310.30	979.89 ± 477.77
CI (L/min/m^2^)	2.79 ± 1.35	2.75 ± 0.7	2.84 ± 0.48	3.63 ± 2.36	2.47 ± 0.58	1.35 ± 1.46	3.01 ± 0.85
MRAP (mmHg)	6.01 ± 4.47	5.82 ± 4.23	5.23 ± 6.15	6.6 ± 4.13	6.56 ± 4.65	6.53 ± 4.30	4.77 ± 4.24
PeakVO2/kg (mL/min/kg)	12.65 ± 3.93	13.89 ± 4.32	11.44 ± 2.66	13.87 ± 4.08	10.55 ± 2.34	10.27 ± 2.37	14.19 ± 4.23
VE/VCO2	42.99 ± 9.80	42.35 ± 9.71	39.96 ± 8.80	42.47 ± 8.13	44.9 ± 11.27	47.03 ± 11.91	40.24 ± 7.47

Continuous variables are presented as mean ± SD. Categorical variables are

given as counts. IPAH, idiopathic pulmonary arterial hypertension; CTD-PAH, pulmonary arterial hypertension associated with connective tissue disease; CHD-PAH, pulmonary arterial hypertension associated with congenital heart disease; CTEPH, chronic thromboembolic pulmonary hypertension; PaO2, arterial oxygen pressure (daytime); PaCO2, arterial carbon dioxide pressure (daytime); SaO2, arterial oxygen saturation (daytime); NT-proBNP, N-terminal pro B type natriuretic peptide; MPAP, mean pulmonary arterial pressure; PVR, pulmonary vascular resistance; CI, cardiac index; MRAP, mean right atrial pressure; PeakVO2/kg, peak kilogram oxygen uptake; VE/VCO2, carbon dioxide ventilation equivalent

### The comparison of sleep breathing monitoring indicators in patients with and without OSA

The AHI boundary value was 5, and 35 (25%) patients had OSA out of the 140 patients with high pulmonary pressure. The AHI in patients with OSA was 16.18 ± 12.65 versus 1.62 ± 1.21 in those without (*P* < 0.001), and the average oxygen saturation was 88.89 ± 4.86 versus 91.90 ± 4.68 (*P* = 0.439). The nocturnal minimum oxygen saturation was 75.8 ± 11.55 versus 83.52 ± 7.13 (*P* = 0.002) (See Table 3).

**Table 3:** Comparison of sleep breathing monitoring indicators in patients with and without OSA

Variable	No OSA	OSA	P value
Numbers	105	35	NS
AHI/h^*^	1.6 ± 1.2	16.2 ± 12.7	< 0.001
AI, /h^*^	0.3 ± 0.5	3.9 ± 9.6	< 0.001
OAI, /h^*^	0.3 ± 0.5	3.3 ± 7.6	< 0.001
CAI, /h	0.0 ± 0.0	0.6 ± 0.5	0.822
Mean SaO2, %^*^	91.90 ± 4.68	88.89 ± 4.86	0.439
Minimum SaO2, %^*^	83.52 ± 7.13	75.8 ± 11.55	0.002
SaO2 < 90%, min^*^	74.97 ± 117.64	166.30 ± 148.17	0.001
SaO2 < 80%, min^*^	9.81 ± 47.68	19.93 ± 62.81	0.085
SaO2 < 70%, min^*^	2.51 ± 25.03	5.21 ± 16.12	0.327

Data are presented as mean ± SD or median (interquartile range). AHI, apnoea and hypopnea index; AI, apnoea index; OAI, obstructive apnoea index; CAI, central apnoea index; mean SPO2, mean oxygen saturation (night); minimum SPO2 minimal oxygen saturation (night)

### The comparison of routine clinical examination values in patients with and without OSA

PH patients with OSA were older (46.74 ± 14.25 years vs. 37.06 ± 12.46, *P* = 0.001) and had a higher proportion of males (57.1% vs. 82.9%, *P* = 0.001) than PH patients without OSA. SBP and DBP were higher in patients with OSA (118.86 ± 14.71 mmHg vs. 111.28 ± 13.22 mmHg, *P* = 0.004 and 76.83 ± 14.79 mmHg vs. 72.26 ± 9.92 mmHg, *P* = 0.006, respectively). PaO2 was lower (63.69 ± 10.21 mmHg vs. 71.77 ± 15.21 mmHg, *P* = 0.014) in patients with OSA than patients without OSA. mPAP was also lower (53.57 ± 13.08 mmHg vs. 56.45 ± 18.19 mmHg, *P* = 0.01) in patients with OSA. The laboratory and haemodynamic characteristics did not differ between the two groups. Echocardiographic and cardiopulmonary exercise test parameters also did not differ between the two groups (see Table 4).

**Table 4:** Comparison of routine clinical examination values in patients with and without OSA

Variable	No OSA	OSA	P value
Numbers	105	35	NS
Age (years)^*^	37.06 ± 12.46	46.74 ± 14.25	0.001
Females (N%)^*^	87 (82.9%)	20 (57.1%)	< 0.001
BMI (kg·m^−2^)	22.32 ± 3.38	25.71 ± 4.13	0.154
SBP (mmHg)	111.28 ± 13.22	118.86 ± 14.71	0.004
DBP (mmHg)	72.26 ± 9.92	76.83 ± 14.79	0.006
NYHA (%)			0.327
1	8 (7.6%)	0 (0%)	
2	46 (43.8%)	8 (22.9%)	
3	47 (44.8%)	26 (74.3%)	
4	4 (3.8%)	1 (2.9%)	
Drug therapy	26/68/11	10/19/6	0.852
Targeting medication (none/single/combination)			
Positive inotropes	79	21	0.206
Diuretics	94	20	0.335
Supplemental oxygen therapy	21	17	0.117
FEV1	78.67 ± 14.87	74.28 ± 13.60	0.783
FVC	86.13 ± 16.75	84.35 ± 14.28	0.671
FEV1/FVC	94.33 ± 7.82	92.66 ± 8.43	0.562
TLC	83.91 ± 9.65	79.37 ± 8.52	0.942
DLCO	72.75 ± 11.68	70.42 ± 14.72	0.923
PaO_2_ (mmHg)^*^	71.77 ± 15.21	63.69 ± 10.21	0.014
PaCO_2_ (mmHg)	35.76 ± 4.74	36.51 ± 11.17	0.843
SaO_2_ (%)	93.24 ± 4.71	91.79 ± 3.77	0.199
Epworth score	3.34 ± 3.12	4.67 ± 2.97	0.740
LA (mm)	30.99 ± 6.27	33.2 ± 6.30	0.4433
LV (mm)	37.36 ± 6.79	41.11 ± 7.36	0.609
LVEF (%)	63.02 ± 5.65	61.97 ± 6.54	0.337
RV (mm)	32.27 ± 6.82	33.8 ± 7.71	0.306
sPAP (mmHg)	88.88 ± 22.90	85.52 ± 17.66	0.321
6MWD (m)	418.66 ± 96.40	368.56 ± 120.62	0.337
NT-proBNP (pg/ml)	1126.31 ± 1298.81	1662.47 ± 1493.71	0.553
mPAP (mmHg)^*^	56.45 ± 18.19	53.57 ± 13.08	0.010
PVR (dyn•s•cm^−5^)	1110.86 ± 517.33	1181.54 ± 402.09	0.071
CI (L/min/m^2^)	3.04 ± 1.34	2.04 ± 1.11	0.730
MRAP (mmHg)	5.97 ± 4.67	6.11 ± 3.89	0.465
Peak VO_2_/kg (ml/kg/min)	13.16 ± 3.95	11.08 ± 3.46	0.272
VE/VCO_2_	42.27 ± 9.21	45.19 ± 11.29	0.380
VE/VCO2 slope	43.33 ± 15.41	48.48 ± 15.19	0.860

Data are presented as mean ± SD or median (interquartile range). BMI, body mass index; SBP, systolic blood pressure; DBP, diastolic blood pressure; NYHA, New York Heart Association; FEV1, forced expiratory volume at first second; FVC, forced vital capacity; TLC, total lung capacity; DLCO, diffusion capacity for carbon monoxide of the lung; PaO2, arterial oxygen pressure (daytime); PaCO2, arterial carbon dioxide pressure (daytime); SaO2, arterial oxygen saturation (daytime); LA, anteroposterior diameter of left atrium; LV, anteroposterior diameter of left ventricle; LVEF, left ventricular ejection fraction; RV, right ventricular end-diastolic diameter; sPAP, pulmonary artery systolic pressure; 6MWD, six-minute walking distance; NT-proBNP, N-terminal pro B type natriuretic peptide; MPAP, mean pulmonary arterial pressure; PVR, pulmonary vascular resistance; CI, cardiac index; MRAP, mean right atrial pressure; PeakVO2/kg, peak kilogram oxygen uptake; VE/VCO2, carbon dioxide ventilation equivalent; VE/VCO2 slope: the slope of carbon dioxide ventilation equivalent

### Logistic regression analysis corrected for BMI to indicate OSA

Through multivariate logistic regression analysis corrected for BMI, we found that age (OR 1.039, 95% CI 1.005–1.975, *P* = 0.025), sex/female sex (OR 0.288, 95% CI 0.112–0.738, *P* = 0.01), SBP (OR 1.03, 95% CI 0.998–1.064, *P* = 0.068), and PaO2 (OR 0.965, 95% CI 0.931–1, *P* = 0.049) were associated with OSA in PH patients. Higher age, male sex, and lower daytime PaO2 had the strongest ability to indicate OSA in PH patients (see Table 5).

**Table 5:** Logistic regression analysis corrected for BMI to indicate OSA

Variable	Odds ratio	95% confidence interval	*P* value
Age (years)	1.039	1.005–1.975	0.025
Females (N%)	0.288	0.112–0.738	0.01
SBP (mmHg)	1.030	0.998–1.064	0.068
PaO2 (mmHg)	0.965	0.931–1	0.049

SBP, systolic blood pressure; PaO2, arterial oxygen pressure (daytime)

## Discussion

Sleep-disordered breathing is an important risk factor for a variety of cardiovascular and metabolic diseases, and it has a high prevalence in patients with PH [15].

OSA is difficult to detect with a routine blood gas analysis on admission because patients often have no typical complaints, such as snoring and daytime drowsiness. Fadia et al. found that sleep apnoea was very common in patients with PH. Their multivariate logistic regression analysis suggested that symptoms did not predict the occurrence of sleep-disordered breathing [16]. In our study, the Epworth score was not significantly different in PH patients with and without OSA. Most clinicians do not perform polysomnography or respiratory polygraphy if the patient does not have the typical complaint of daytime sleepiness, but this may result in a significant underestimation of OSA incidence. Very few OSA patients have daytime drowsiness, which may be associated with the high sympathetic activity in OSA [17].

Ulrich et al. analysed 38 patients with PH, mainly IPAH, CHD-PAH and CTEPH. When AHI ≥ 10/h was defined as the cut-off value, 11% of the patients with PH presented OSA [18]. Florian et al. showed that sleep-disordered breathing was common in PAH and CTEPH patients and reflected disease severity [19]. Our study demonstrated a high incidence of OSA in patients with PH, as OSA was present in 25% (35/140) of them, which is higher than found abroad. At the same time, the use of oxygen therapy, diuretics, and targeted drugs for PH may alleviate OSA symptoms in these patients, so the incidence of OSA may still be underestimated. The differences between the studied patient populations may account for the differences in the results.

When we compared patients with and without OSA, older males were more likely to have OSA, which is consistent with earlier studies. A unique finding of our study was that a decrease in the partial pressure of arterial blood oxygen during the day could indicate an increased risk of OSA in PH patients.

When comparing the relationships between different types of PH and OSA, we found that OSA had the highest incidence in patients with lung disease– or hypoxia-related PH (76%); in CTEPH patients, the incidence of OSA was 50%. The presence of OSA in patients with CTEPH is not clearly explained, but current studies report a high incidence of OSA in patients with acute pulmonary embolism and deep vein thrombosis [20, 21].

According to our study, it is not yet possible to quantify the correlation between OSA and the severity of PH. Our study did not find that PH patients who also had OSA had more severe heart damage signs, including BNP, 6MWD, and CI. Minai et al. performed nocturnal oximetry tests on 43 patients with IPAH and CTD-PAH and found that the sleep-disordered breathing group had a higher BNP, higher mRAP, higher mPAP, higher PVR, and lower CI than the no-OSA group, suggesting that sleep-disordered breathing is associated with the progression of PH and the dysregulation of right ventricular function [22]. In our study, only mPAP showed the same pattern; that is, in PH patients with OSA, mPAP was significantly lower than that in patients without OSA, while we found no significant differences in other haemodynamic indicators. We also found no significant difference in any of the echocardiographic indices between the two groups. It cannot be concluded that OSA is related to the severity of PH, the destruction of cardiac structure or the decline of function. We also compared the CPET indicators between the two groups of patients and found no significant difference. Whether the patients had OSA did not have a significant impact on the cardiopulmonary exercise capacity of these PH patients.

Regarding the link between sleep-disordered breathing and pulmonary hypertension, the mechanism by which OSA influences the left heart is complicated. First, hypoxia causes pulmonary vasoconstriction, and pulmonary vascular resistance increases, leading to an increase in precapillary pulmonary artery pressure. In addition, during an obstructive event, the increases in chest negative pressure, venous return, right ventricular preload and stroke volume lead to an increase in pulmonary artery blood flow, which can also lead to an increase in pulmonary artery pressure. At the same time, OSA can increase the afterload of the patient's left ventricle by increasing the transmural pressure of the left ventricle and the negative pressure in the chest cavity. These factors can damage the patient's left heart function and cause an increase in pulmonary venous pressure [23].

Sajkov et al. [24] showed that PH associated with obstructive sleep apnoea is usually mild to moderate, can be reversed by positive airway pressure therapy, and is usually associated with significant hypoxic vascular reactivity (i.e., pulmonary artery pressure and lack of oxygen exposure). In patients with fibrotic lung disease, including that which may occur in scleroderma, pulmonary artery pressure may increase due to hypoxic pulmonary vasoconstriction and destruction of the lung parenchyma. Pulmonary artery expansion does not necessarily correspond to increased pulmonary artery pressure, but expansion is related to sleep-disordered breathing [25, 26]. In patients with systemic sclerosis, we should pay close attention to the association between sleep-disordered breathing and pulmonary artery dilation [27]. About parenchymal lung disease, especially chronic obstructive pulmonary disease (COPD), an important paper in the New England Journal of Medicine suggests that dilation of the pulmonary arteries may indicate the worsening of COPD [28]. We want to know whether OSA has an important causal relationship in this association, especially considering recent evidence that positive airway pressure can reduce the risk of rehospitalization in patients with frequent COPD worsening [13]. As mentioned above, the definition of PH has recently changed (i.e., the mean pulmonary arterial pressure is 20 mmHg instead of 25 mmHg), suggesting that earlier studies may have underestimated the true burden of PH. Therefore, we highly support further efforts to explore the link between PH and OSA, especially in patients with pulmonary parenchymal disease. Our study did not investigate whether PH could be alleviated by the correction of OSA. Large, prospective cohort studies are needed to further explore these issues.

## Conclusion

OSA has a high incidence in patients with PH. OSA may aggravate PH to some extent, and advanced age, male sex and lower daytime PaO2 could predict the presence of OSA. It may be important to identify and treat PH patients with OSA. The clinical significance of PH with OSA requires further investigation.

### Limitation

First, because polysomnography is time-consuming and laborious, it is generally not used for preliminary screening. Therefore, portable cardiopulmonary breathing monitoring was used for patient sleep monitoring in this study. Due to the low incidence of PH, too few patients had each disease type for subgroup analysis, and selection bias was inevitable in this prospective study. In addition, we did not follow up the patients, so we could not determine the effect of OSA on the long-term survival of patients with PH. We did not investigate whether PH could be alleviated by the correction of OSA. Large, prospective cohort studies are needed to further explore these issues.

## Data Availability

All relevant raw data, will be freely available to any researcher wishing to use them for non-commercial purposes, without breaching participant confidentiality.
